# 

**Published:** 1899-10

**Authors:** 


**NOTICE.—This JOURNAL and THE INTERNATIONAL JOURNAL OF SURGERY one year for $1.00 cash, to new subscribers. No out-of-town checks accepted unless 10c. is added for cost of cashing.**

***NOTICE.—This JOURNAL and THE INTERNATIONAL JOURNAL OF SURGERY one year for $1.00 cash, to new subscribers. No out-of-town checks accepted unless 10c. is added for cost of cashing.***

### TO SUBSCRIBERS.

**Those who are in arrears for subscriptions to Atlanta Medical and Surgical Journal get statement this month on bill-head of Atlanta Journal-Record of Medicine. Those in arrears to Southern Medical Record will get statement on "Record" bill-head. All remittances should be made to Atlanta Journal-Record of Medicine. We have employed a careful accountant to make out these bills, but are ready to correct any error. The present subscription price, $1.00, is so small that no one should fail to pay his bill promptly.**

***NOTICE.—This JOURNAL and THE INTERNATIONAL JOURNAL OF SURGERY one year for $1.00 cash, to new subscribers. No out-of-town checks accepted unless 10c. is added for cost of cashing.***

